# High Frequency of Fibromyalgia in Patients with Psoriatic Arthritis: A Pilot Study

**DOI:** 10.1155/2013/762921

**Published:** 2013-02-14

**Authors:** Marina N. Magrey, Maria Antonelli, Neena James, Muhammad Asim Khan

**Affiliations:** MetroHealth Medical Center, Case Western Reserve University School of Medicine, 2500 MetroHealth Drive, Cleveland, OH 44109, USA

## Abstract

*Background.* Widespread pain from fibromyalgia syndrome (FMS) is observed in patients with psoriatic arthritis (PsA). We hypothesized that there is increased frequency of FMS in patients with PsA that contributes to fatigue and pain. *Method.* We prospectively enrolled patients with PsA based on the Classification criteria for Psoriatic Arthritis and healthy subjects were used as controls. The frequency of FMS was determined using London Fibromyalgia Epidemiologic Study Screening Questionnaire (LFESSQ) and Symptoms Intensity scale (SIs). *Results. *34 PsA patients and 44 controls fulfilled the inclusion criteria. Median age of PsA patients was 52 years with 53.33% females. Median age of controls was 50.5 years with 59% females. FMS was present in 53.33% of PsA patients compared to 4.54% of the controls (*P* < 0.001), based on LFESSQ. 37.50% of PsA had FMS compared to 6.66% of controls (*P* < 0.001) based on SIs. There was a significant correlation between LFESSQ and SIs in the psoriatic group (*P* = 0.00243). 76.66% of PsA patients complained of fatigue compared to 40.90% of controls, but the mean fatigue score between the two groups was comparable (5.03 versus 5.18). *Conclusion. *FMS-associated pain and fatigue are significantly more frequent in patients with PsA compared to controls.

## 1. Introduction

Chronic inflammatory arthritis which includes diseases such as rheumatoid arthritis (RA), psoriatic arthritis (PsA), ankylosing spondylitis (AS), and other forms of spondyloarthritis (SpA) are characterized by joint inflammation leading to joint destruction. Despite significant progress in the treatment of joint inflammation with the availability of biologics, pain that is either related or unrelated to disease activity persists in many patients causing substantial impairment of physical function and quality of life [1, 2]. Chronic widespread non-articular pain from fibromyalgia syndrome (FMS) is observed in patients with several rheumatic diseases: rheumatoid arthritis (57%), systemic lupus erythematosus (40%), and Sjögren's syndrome (47%) [3–5]. FMS has also been recently noted in patients with Granulomatosis with Polyangiitis [6]. Pain from FMS has been thought to be secondary to central sensitization [4]. Such pain processing may be due to dysfunctional nervous system responses to physical or psychosocial stressors [7, 8]. Treatment is centered on pain control and behavioral changes that are mostly supportive. 

Fibromyalgia seen in patients with inflammatory arthritis has been controversially termed secondary fibromyalgia and attributed to underlying inflammation. Proinflammatory cytokines like TNF-*α* have been implicated in development of abnormal central pain responses in animal studies [9, 10]; however, the link between pain and inflammation is not well established in human studies. In a recent study of early inflammatory arthritis, objective measures of inflammation, such as erythrocyte sedimentation rate, C-reactive protein and swollen joint count, were not associated with the clinical diagnosis of FMS, suggesting that inflammation does not predict FM diagnosis [11]. Hence, fibromyalgia can be considered a comorbidity in inflammatory arthritis.

Since the pathophysiology and treatment of PsA differ greatly from FMS, it is imperative to differentiate between these two conditions to direct appropriate treatment at symptoms of these often comorbid conditions. The purpose of our pilot study was to determine the frequency of FMS in patients with PsA as compared to age-matched healthy controls. We hypothesized that there is an increased frequency of FMS in patients with PsA that contributes to fatigue and pain. 

## 2. Materials and Methods

This pilot prospective study was approved by the institutional review board of our local institute. The patients were enrolled between 1/2009 and 12/2009 from our Rheumatology Outpatient Clinic. Healthy subjects who were seen for annual physical examination in the Internal Medicine Clinic were used as controls. All consecutive patients who consented to the study were enrolled. Two validated instruments were used to measure the frequency of fibromyalgia: the London Fibromyalgia Epidemiologic Study Screening Questionnaire (LFESSQ) and Symptoms Intensity scale (SIs). The LFESSQ is a questionnaire commonly used to screen for FMS in the general population [12]; this is a 6-item instrument with 4 items related to pain and 2 items related to fatigue. A positive screen was defined as all 4 items positive for pain. (Pain in all 4 items and on both sides of the LFESSQ was used to identify FMS.) This 6-item instrument has a sensitivity of 93.5% in FMS patients and specificity of 80.0% in RA patients [8]. The SIs consists of pain in 19 paired non-articular regions, also called Regional Pain Scale (RPS) combined with a fatigue score of 0–10 on fatigue visual analog scale (FVAS) [13–15]. A score ≥8/19 on RPS and a fatigue score ≥6 show moderate concordance with the American College of Rheumatology criteria for FMS [16]. Furthermore, the SIs (FVAS + RPS/2)/2 (range 0–9.5) shows better correlation with general health using global measures than the Health Assessment Questionnaire and has better correlation with death, cardiovascular disease, and myocardial infarction [14]. These instruments offer another particular advantage of determining frequency of FMS without the need for physical examination. The SI scale ((fatigue VAS + RPS/2)/2) with a cut off 05.75 (range 0–9.5) differentiates fibromyalgia from other painful conditions [17]. It is an effective measure of fibromyalgia symptom intensity and prevalence.

English speaking patients (pts) with PsA based on Classification criteria for Psoriatic Arthritis (CASPAR) [18] and controls with age ≥18 years were consented and enrolled. Questionnaires were administered to consecutive PsA pts and healthy controls at the time of their visit.

Statistical analysis was done with categorical measures summarizing frequencies and percents. A Pearson's correlation was used to look at relationship between 2 variables.

## 3. Results

A total of 78 patients were included in the study: 34 PsA pts and 44 controls.  Table 1 describes the demographics and cohort descriptions. Median age of PsA patients was 52 years (range 30–70) with 53.33% females. Median age of controls was 50.5 years (range 44–56) with 59% of females. 

**Figure 1:**
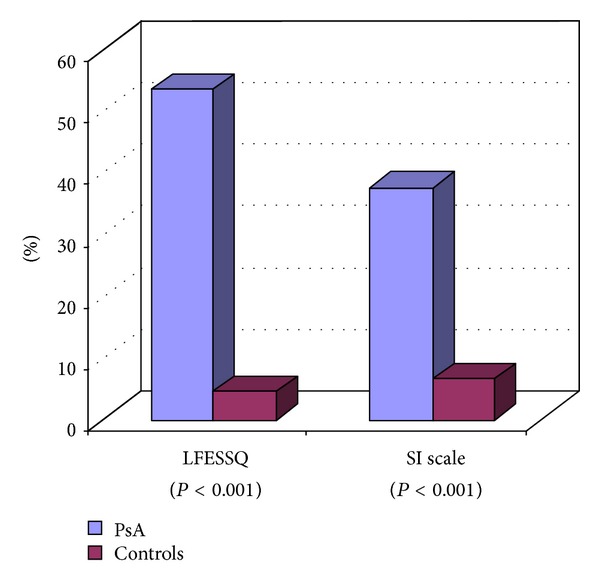
There was a correlation between the scores of LFESSQ and SIs in the psoriatic group with *P* value = 0.00243.

**Figure 2:**
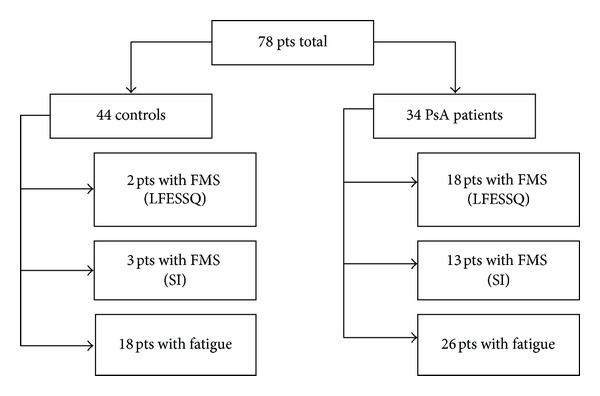
Consort figure describing the number of patients total in the study and breakdown of patients in each group with FMS based on LFESSQ or SI and patients with fatigue.

The frequency of FMS documented in controls and PsA patients is described in Table 2. FMS was present in 53.33% of PsA patients compared to 4.54% of the controls (*P* < 0.001), based on LFESSQ. 37.50% of PsA had FMS compared to 6.66% of controls (*P* < 0.001) based on SIs. There was a correlation between the scores of LFESSQ and SIs in the psoriatic group with *P*-value = 0.00243. 76.66% of PsA complained of fatigue compared to 40.90% of controls, but the mean fatigue score between the two groups was comparable (5.03 versus 5.18) (Figure 1).  Figure 2 is a depiction of the breakdown of each patient enrolled into the study.

## 4. Discussion

We report a statistically higher frequency of fibromyalgia (37.5%) among 34 patients with PsA as compared to healthy controls (6.7%). At present there are no studies regarding the prevalence of fibromyalgia in PsA except a previous study that has reported the frequency of 10 or more fibrositic tender points in patients with PsA to be 24% [19]. That study used clinical assessment with palpation and dolorimeter scores to distinguish between articular and non-articular pain. This prior study has alluded to an association between FMS and PsA. A recent study showed only 6.9% of patients with PsA having at least 11 tender points upon examination [20]. Our pilot study is the first to document the frequency of FMS in patients with PsA using standardized instrument measures. 

The reported prevalence of FMS in general population is between 2% and 4% [21–23]. However, the frequency of fibromyalgia in our controls was high at 6.7%. Making an adjustment for increased frequency of FMS in our patient population, the adjusted frequency of FMS in our PsA patients could be 22% which is still higher than the general population.

Chronic fatigue syndrome (CFS) is frequently seen in patients with FMS and patients who meet the criteria for both FMS and CFS have a worse overall health status [24]. Higher fatigue scores have been reported in patients with PsA [20, 25] and are related to patient-reported assessments of disability, pain, and psychological distress. Our results confirmed these reports with 76% of patients with PsA having fatigue. Effective management of fatigue in PsA patients may be ameliorated by identifying and treating underlying FMS. 

The findings in this study and future potential studies surrounding this issue have important implications regarding patients with PsA. Patients with PsA with high disease activity status and poor functionality may benefit from fibromyalgia screening. If patients with PsA have chronic diffuse pain and fatigue attributed to FMS, these patients will require multidisciplinary management strategies, including concurrent pharmacologic treatments and nonpharmacologic therapy like aerobic exercise and cognitive behavioral therapy [26]. 

We used the new symptom-based diagnostic criteria for FMS comprising of regional pain scale and a visual analogue scale for fatigue. The Symptom Intensity scale is a diagnostic tool as well as a simple measure of general health among all rheumatic disease patients [27]. It was developed by Wolfe based on survey mailed to 12,799 patients who had RA, osteoarthritis, or FMS. The respondents were asked if they had pain in 38 articular and non-articular regions and also to complete 10 cm visual analog scale. He observed that fibromyalgia could be differentiated from other 2 diseases based on pain primarily in 19 non-articular sites which he called RPS. Wolfe also showed that a score of at least 8 points on RPS, combined with a core of at least 6 cm on visual analogue provided the best diagnostic precision consistent with FMS [13]. The combination of these 2 measures latter came to be known as Survey Criteria [14]. 

These criteria can be used for large scale clinical and epidemiological studies in which clinical examination is not feasible. 

There may be an overlap between the clinical features of PsA and FMS particularly in patients with enthesitis. We did not study the enthesitis involvement in PsA patients as there is no validated instrument to study it. The power of Doppler ultrasonography to detect enthesitis is not specific for PsA and entheseal inflammation was seen in 21% of patients with FMS [20].

In conclusion, FMS-associated pain and fatigue are significantly more frequent in patients with PsA compared to controls, as shown by our pilot study. There is a need to perform a study based on larger number of patients to confirm these findings. We suggest that all PsA patients with chronic and persistent pain and fatigue should also be evaluated for FMS before initiating treatment because of common concurrence of the two conditions. 

## Figures and Tables

**Table 1:** Demographics and cohort descriptions.

	Controls	Patients with PsA
Total patients, number	44	34
Men, number (%)	18 (41)	16 (47)
Women, number (%)	26 (59)	18 (53)
Median age years (range)	50.5 (44–56)	52 (30–70)

**Table 2:** Frequency of FMS documented in controls and PsA patients.

	Controls	Patients with PsA	*P* value
FMS, number (%) based on LFESSQ	2 (4.5)	18 (53.3)	<0.001
FMS, number (%) based on SI	3 (6.6)	13 (37.5)	<0.001
Fatigue, number (%)	18 (40.9)	26 (76.6)	
